# Treatment Implications: Using Neuroscience to Guide the Development of New Pharmacotherapies for Alcoholism

**Published:** 2008

**Authors:** Suchitra Krishnan-Sarin, Stephanie O’Malley, John H. Krystal

**Keywords:** Alcoholism, alcohol and other drug dependence, treatment, neurochemical systems, neuropharmacology, drug therapy, pharmacotherapy, disulfiram, naltrexone, acamprosate, selective serotonin reuptake inhibitors (SSRIs), topiramate, ondansetron, baclofen, antipsychotics, animal models, human studies

## Abstract

Developing pharmacotherapies to treat alcohol dependence and associated health problems traditionally has been based on gaining a better understanding of the neuroscience underlying alcohol-drinking behavior. To date, three medications have been approved for the treatment of alcohol dependence: disulfiram (Antabuse^®^), naltrexone (Revia^®^, Vivitrol^®^, and Naltrel^®^), and acamprosate (Campral^®^). However, these medications have modest efficacy, and there is a great need for newer medications that target different neurochemical systems and which could be used either as adjunctive treatments or to treat subpopulations of drinkers. Furthermore, it also is important to improve current treatment options by understanding and incorporating differences in how people with certain genes respond to medication (i.e., pharmacogenetic differences).

Alcohol dependence and its associated health problems are the third leading cause of morbidity and mortality in the United States. Alcohol dependence is a chronic relapsing disorder and is optimally treated using a combination of psychosocial and pharmacological treatments. Most of the work on medication development to date has focused on four primary areas: treating withdrawal symptoms, reducing consumption of and craving for alcohol, preventing relapse, and treating associated psychiatric problems. Discussion of the treatment of withdrawal from alcohol, which involves a combination of pharmacologic therapy and nutritional and psychosocial support, is beyond the scope of this review. This article focuses on therapeutic agents that reduce alcohol drinking and craving and prevent relapse. These agents were developed using animal models—or a translational approach to understanding the mechanisms of how alcoholism arises and persists from a molecular, neurochemical, and behavioral perspective. Importantly, animal models that assess a wide range of alcohol-related behaviors, including drinking, dependence, craving, and relapse, have provided an essential foundation for much of this translational research (see reviews by Egli 2005; Lovinger and Crabbe 2005).

Alcohol-drinking behavior is mediated through complex interactions between mechanisms underlying the reinforcing effects of alcohol. Moreover, the positive reinforcing effects of alcohol are mediated through complex interactions between multiple neurochemical systems. These systems target the cortico-mesolimbic dopaminergic pathway,^1^ which extends from the ventral tegmental area to the nucleus accumbens and has been shown to be important in the rewarding effects of many drugs, including alcohol. This pathway is indirectly activated by alcohol through the release of other neurotransmitters, including opioids, serotonin, glutamate, γ-aminobutyric acid (GABA), and acetylcholine. Following chronic use of alcohol, many of these same neurochemical systems undergo adaptations and attempt to achieve homeostasis when alcohol is withdrawn, which then leads to alcohol withdrawal symptoms and consumption of alcohol for negative reinforcement, or avoidance of withdrawal. Most medications in use or in development for alcoholism treatment act on these neurotransmitter systems (see figure) and, in many instances, are focused on normalizing the alcohol-specific neuroadaptations or blocking alcohol-specific reinforcement. Recent efforts to develop new medications have focused on nonspecific neural mechanisms mediating alcohol drinking, such as stress and motivation/self-control.

Three drugs currently are approved by the U.S. Food and Drug Administration (FDA) for the treatment of alcoholism: disulfiram (Antabuse^®^), naltrexone (Revia,^®^ Vivitrol,^®^ and Naltrel^®^), and acamprosate (Campral^®^). The following sections will describe each of these agents, including their efficacy and the neuroscience underlying treatment response. The article also will discuss other agents that have shown some promise in reducing drinking and finally present preliminary evidence from some exciting investigational agents that still are under evaluation.

## FDA-Approved Treatments for Alcoholism

### Disulfiram

Disulfiram has been in use for the treatment of alcoholism since the 1940s. This medication produces an aversive effect by disrupting alcohol metabolism. When alcohol is consumed, it is converted to acetaldehyde, which is further broken down by aldehyde dehydrogenase. Disulfiram inhibits aldehyde dehydrogenase, which leads to an excessive buildup of acetaldehyde and results in many unpleasant effects, including lowered blood pressure, palpitations, nausea, vomiting, headache, and difficult breathing. Anticipation of these aversive effects can be used to discourage drinking.

Although the proposed mechanism of action of disulfiram on alcohol use has been thought to be primarily related to the inhibition of liver aldehyde dehydrogenase, its efficacy also could be related to secondary central nervous system actions, through modulation of catecholamine neurotransmission. Specifically, at clinical doses, disulfiram inhibits the enzyme dopamine–β–hydroxylase, which converts dopamine to norepinephrine, potentially leading to increases in dopamine levels (Goldstein and Nakajima 1967; Goldstein et al. 1964).

Clinical trials with disulfiram have found lower rates of relapse to drinking in those who are compliant with the medication (Fuller et al. 1986). However, because of the aversive nature of this therapy, noncompliance is one of the biggest problems with its use. The use of this medication is supervised in many clinical settings. Additionally, side effects including hepatotoxicity, depression, and psychotic reactions limit the use of this medication.

Preliminary evidence suggests that disulfiram may have some benefit in reducing alcohol craving and increasing consecutive days of abstinence among alcoholics with comorbid psychiatric disorders (Petrakis et al. 2005) and in reducing cocaine and alcohol use among cocaine users (Carroll et al. 1998). Thus, disulfiram may have particular value in patients who abuse alcohol in the context of depression or polysubstance abuse.

### Naltrexone

Naltrexone is available as an oral medication (Revia^®^) and in two injectable forms (Vivitrol^®^ and Naltrel^®^). Its primary use is for the treatment of alcohol dependence, and it is well tolerated with primarily gastrointestinal side effects (O’Malley et al. 1992; Volpicelli et al. 1992). Naltrexone’s efficacy in reducing alcohol drinking is believed to be mediated through interactions between the endogenous opioid system and dopamine systems, specifically through antagonism of the μ–opioid receptors. Evidence from animal models indicates that alcohol increases release of β–endorphins in regions of the brain known to be involved in alcohol reward and that naltrexone administration blocks this release (Marinelli et al. 2003; Zalewska-Kaszubska et al. 2006). Moreover, naltrexone and similar opioid antagonists have been shown to reduce drinking in a variety of animal models (e.g., see reviews by Froehlich et al. 2003; Swift 2000). Clinical trials indicate that treatment-seeking drinkers who receive naltrexone at a dose of 50 mg/day in combination with a behavioral intervention have lower levels of relapse to drinking during the treatment period. This effect of naltrexone may be related to a reduction in alcohol craving (e.g., O’Malley et al. 2002).

It is important to note, however, that not all clinical trials conducted with naltrexone over the past decade have observed significant improvements in drinking-treatment outcomes (e.g., Krystal et al. 2001). Two recent meta-analytical reports (Bouza et al. 2004; Srisurapanont and Jarusuraisin 2002) suggest that naltrexone has modest efficacy in preventing relapse to drinking. Moreover, although naltrexone is relatively well tolerated, the potential risk of hepatotoxicity at high doses requires caution when treating patients with liver disease. Moreover, because it is an opiate antagonist, it is contraindicated in alcoholics who also use opiates.

A more recent multisite trial (Anton et al. 2006) that used a higher dose of naltrexone (100 mg/day) with medical management to enhance compliance found that this treatment significantly reduced alcohol use, although the effect size was small. The efficacy of naltrexone was stronger in patients who did not receive cognitive–behavioral therapy in addition to medical management (Anton et al. 2006). A newer, extended-release formula of naltrexone has shown similar efficacy. In a 6-month trial, rates of heavy drinking were significantly lower in patients receiving a 380-mg dose, with a greater effect observed in those who achieved abstinence prior to initiating treatment (Garbutt et al. 2005). This injectable long-acting formula appears to produce more stable blood levels than the oral medication (i.e., it reduces peak levels that might be associated with side effects and elevates trough levels, which should improve efficacy).

One hypothesis that might reconcile the modest efficacy of naltrexone in large clinical trials with the more robust findings in some smaller single-site studies would be if there was a subpopulation of alcohol-dependent patients who were naltrexone responsive among a larger population of naltrexone-nonresponsive patients (e.g., see Gueorgueiva et al. 2007). Subpopulations, may be defined, for example, by genetic approaches. Specifically, alcoholics with a family history of alcoholism appear to have greater reductions in alcohol consumption with naltrexone compared with those who have no family history of alcoholism (Krishnan-Sarin et al. 2007). In addition, people with a variant of the gene for the μ-opioid receptor where naltrexone produces its effects (i.e., the *OPRM1* gene) respond better to naltrexone treatment in some trials (Anton et al. 2008; Oslin et al. 2003) but not in others (Gelernter et al. 2007). Although it still is unclear whether this genotypic difference reflects a type of patient or is related to the reaction to naltrexone, these kinds of studies raise the issue of treatment matching by genetic background. For example, it is possible that some patients may need low doses of naltrexone because of relative intolerance or side effects, whereas others may need higher doses to achieve clinical efficacy.

### Acamprosate

Acamprosate is available in an oral, delayed-release formula called Campral^®^. Researchers have proposed that acamprosate’s actions may be mediated through antagonism of the *N*-methyl d-aspartate (NMDA) glutamate receptor site or via modulation of glutamate neurotransmission at metabotropic-5-glutamate receptors (De Witte et al. 2005; Harris et al. 2002). Acamprosate has been shown to reduce neuronal hyperexcitability during withdrawal from alcohol (Spanagel et al. 1996), possibly because of reductions in glutamate levels (Dahchour et al. 1998), which may help to normalize the balance between excitatory and inhibitory pathways produced by chronic alcohol use (Littleton and Zieglgansberger 2003). However, recent findings (Reilly et al. 2008) suggest that these hypothesized mechanisms of action are not evidenced in the therapeutic dose range that normally is used to reduce alcohol use.

Nevertheless, acamprosate reduces both alcohol use in animal models (e.g., Czachowski et al. 2001) and responses to alcohol cues in alcohol-dependent animals (Spanagel et al. 1996). Acamprosate’s efficacy in reducing alcohol use in alcohol-dependent drinkers was first established in Europe through multiple randomized controlled trials. A meta-analysis (Mann et al. 2004) of 17 of these studies found a modest, but significant, benefit for acamprosate in improving continuous abstinence from alcohol at 6 months. More recent evidence (Kranzler and Gage 2008) using combined data from three pivotal European trials replicated these findings and found that rates of complete abstinence as well as percent days abstinent and time to first drink all were significantly greater with acamprosate treatment. Acamprosate is well tolerated, with patients reporting only minimal side effects, primarily gastrointestinal, in most clinical trials. Finally, because acamprosate has been shown to reduce alcohol withdrawal in animals, it also may have some benefit as an adjunctive treatment in alcohol detoxification.

In contrast to the European findings, the intent-to-treat analyses from two multisite studies conducted in the United States indicate no benefit for acamprosate when compared with placebo in reducing alcohol use in treatment-seeking alcoholics (Anton et al. 2006; Mason et al. 2006). The reason for these discrepancies between the U.S. and European trials still is unclear, but it has been proposed that the higher level of psychosocial intervention provided by the U.S. trials may have masked the efficacy of acamprosate or that fewer heavily dependent patients were enrolled in the U.S. studies. Moreover, one of the trials (Mason et al. 2006) also observed that patients with a treatment goal of abstinence, who were medication compliant, were observed to significantly benefit from the use of acamprosate.

Finally, it also is possible that these discrepancies may be mediated by genetic differences in the populations examined. For example, exciting emerging evidence suggests that alcohol effects are altered in mice carrying various mutations of the glutamatergic genes (see review by Gass and Olive 2008). If the effects of acamprosate are indeed mediated through this glutamatergic system, it would be worth examining these genetic markers as mediators of treatment response. Finally, patient-specific treatment matching also may enhance acamprosate’s efficacy. Evidence from a pooled analyses of seven European trials suggests that alcoholics with increased levels of anxiety, negative family history, and late age of onset of alcoholism, as well as those who are women, may benefit from this medication (Verheul et al. 2005).

## Other Promising Medications With Some Clinical Evidence of Efficacy

Although the agents reviewed in this section are not FDA approved for treating alcoholism, they show promise for this purpose.

### Topiramate

Topiramate, an antiseizure medication, has shown efficacy in reducing alcohol use in recent clinical trials. Topiramate’s actions have been associated with antagonism of α-amino-3-hydroxy-5-methylisoxazole-4-propionic acid (AMPA) and kainate glutamate receptors as well as inhibition of extrasynaptic GABA_A_ receptors, l-type calcium channels, and voltage-dependent sodium channels. In animal models, topiramate has been shown to reduce alcohol use (Nguyen et al. 2007) and alcohol withdrawal-induced convulsions (Farook et al. 2007).

Clinical trials with topiramate in doses of 200 to 300 mg/day have been shown to reduce the percentage of heavy-drinking days (for review, see Johnson et al. 2008). These studies did not require abstinence prior to initiation of topiramate but rather investigated whether topiramate would reduce the frequency of heavy drinking and increase abstinent days over time. Consistent with this approach, recent evidence also suggests that topiramate is more effective than placebo, and is not distinguishable from diazepam, when used for the treatment of alcohol withdrawal symptoms (Krupitsky et al. 2007). One potential limitation of topiramate is its side effect profile, which includes numbness, anorexia, cognitive difficulty, and taste distortion, as well as some rare incidents of visual side effects including myopia, glaucoma, and increased intraocular pressure. Most human clinical trials to date have used a slow titration over several weeks to the desired dose in order to reduce the incidence of side effects.

### Selective Serotonin Reuptake Inhibitors

The use of selective serotonin reuptake inhibitors (SSRIs) in the treatment of alcohol drinking is based on an extensive preclinical literature which shows that lowering brain serotonin levels decreases preference for alcohol (Myers and Veale 1968) and that SSRIs suppress alcohol consumption (e.g., Gulley et al. 1995). These preclinical results have led to a number of clinical trials on the use of SSRIs for treating alcohol use. However, the results of these trials, which have been conducted with medications such as fluoxetine, citalopram, and sertraline, are inconclusive (e.g., Gorelick and Paredes 1992; Kranzler et al. 1995; Naranjo et al. 1995) and do not support the use of SSRIs for treatment of alcohol use. Considering the efficacy of SSRIs in the treatment of depression, few clinical trials also have examined the utility of these agents in reducing alcohol use in depressed alcoholics. Evidence from such trials is inconsistent, however (Cornelius et al. 1997; Kranzler et al. 2006).

It is important to point out that the efficacy of these agents in reducing alcohol use may be genetically mediated. Specifically, it has been observed that type A or late-onset alcoholics may benefit more from the use of SSRIs than type B or early-onset alcoholics (Kranzler et al. 1996; Pettinati et al. 2000). These results suggest that the utility of SSRIs for the treatment of alcohol dependence could be improved by treatment matching.

### Ondansetron

The use of the serotonin-3 (or 5-HT_3_) receptor antagonist ondansetron, an antinausea medication, in the treatment of alcohol use also emerges from extensive preclinical literature which suggests that alcohol exerts effects through the 5-HT_3_ receptors in the brain and that these receptors may mediate the reinforcing effects of alcohol (see review by Barnes and Sharp 1999). Human laboratory studies (Johnson et al. 1993; Swift et al. 1996) have found that ondansetron decreases alcohol preference and desire to drink. Clinical trials of this medication have shown efficacy in reducing drinking behavior, especially in drinkers with early-onset (type B) alcoholism (Johnson et al. 2000; Kranzler et al. 2003). Interestingly, serotonin agonists also have been shown to have alcohol-like effects in people with early- onset alcoholism. Taken together, these findings raise the possibility that the efficacy of 5-HT_3_ agents may be mediated by pharmacogenetic differences.

### Baclofen

Baclofen, a GABA_B_ receptor agonist used clinically for the treatment of muscle spasticity, has been shown in preclinical trials to decrease alcohol intake (Colombo et al. 2004). Results from two small clinical trials (Addolorato et al. 2002; Flannery et al. 2004) indicate that when this medication is used in a dose of up to 30 mg/day, it has minimal side effects and improves drinking outcomes. A more recent clinical trial (Addorolato et al. 2007) in alcohol-dependent patients with cirrhosis suggests that this agent is well tolerated and has some efficacy in improving abstinence rates. More clinical research with this promising agent is needed to establish its efficacy and tolerability in alcohol drinkers.

### Atypical Neuroleptics

Atypical antipsychotics such as aripiprazole and quetiapine act on the dopaminergic and serotonergic systems and have unique binding profiles. Specifically, they antagonize the D_2_ dopaminergic receptor under hyperdopaminergic conditions and act as agonists at the same receptor under hypodopaminergic conditions. The unique binding profile of these agents increases their tolerability when compared with pure D_2_ receptor antagonists like haloperidol. Because the dopaminergic system is involved in alcohol’s reinforcing effects, agents that act on this system should have some efficacy in reducing alcohol’s subjective effects.

Aripiprazole has been shown in preclinical studies to reduce alcohol drinking (Ingman et al. 2006), but the evidence supporting the clinical efficacy of this agent is inconclusive. This drug has a complicated pharmacology that may distinguish it from other currently available antipsychotic medications. In particular, it is an antagonist at 5-HT_6_ receptors, a low-activity (antagonist-like) partial agonist at dopamine D_2_ and serotonin 5-HT_2A_ and 5-HT_7_ receptors, and a high-activity partial agonist (agonist-like) at dopamine D_3_, D_4_, 5-HT_1A_, and 5-HT_2C_ receptors (Shapiro et al. 2003). The use of this agent in a double-blind, placebo-controlled trial, at a maximal dose of 30 mg/day (titrated up from 2 mg/day), resulted in a high rate of side effects, dropouts, and no improvement in alcohol outcomes (Anton et al. 2008). In contrast, in a human laboratory study in which this medication was used in a lower dose of 2.5 to 10 mg/day, it was relatively well tolerated and reduced alcohol’s subjective effects (Kranzler et al. 2008).

There is slightly more clinical evidence supporting the use of quetiapine for alcoholism treatment. Two small placebo-controlled clinical trials with quetiapine suggest that this agent increases rates of abstinence in treatment-seeking alcoholics and that it might be especially useful in those with early-onset and more severe alcoholism (Kampman et al. 2007; Monnelly et al. 2004).

## New Directions and Investigational Agents

The agents reviewed below currently are under investigation and represent new directions for treating alcohol use. Unlike those reviewed above, the following agents have no clinical evidence of efficacy for treating alcoholism.

### CB-1 Receptor Antagonists

The endogenous cannabinoid system recently has been shown to have a role in alcohol’s reinforcing effects, possibly via interaction with the mesolimbic dopaminergic system. Specifically, alcohol intake has been observed to increase levels of endogenous cannabinoids like anandaminde and 2-arachidonylglyc-erol and to downregulate cannabinoid-1 (CB-1) receptors. Preclinical studies indicate that CB-1 receptor antagonists reduce alcohol drinking (Dyr et al. 2008) and that mice which lack the CB-1 receptor consume less alcohol (Colombo et al. 2002) and have increased alcohol sensitivity and withdrawal (Naassila et al. 2004). A recently completed placebo-controlled, double-blind trial with the CB-1 antagonist rimonabant (Soyka et al. 2008), at a dose of 20 mg/day, found that the drug was well tolerated with minimal side effects but that there was no observable improvement in alcohol treatment outcomes. In addition, this medication was found to increase thoughts of suicide in smokers, which may limit its use for the treatment of alcohol dependence.

### Nicotinic Agonist, Partial Agonists, and Antagonists

Preclinical evidence suggests that nicotinic receptors may be involved in alcohol reinforcement, self-administration (Lê et al. 2003), and mediating alcohol cues (Lof et al. 2007). Based on this evidence, researchers have investigated the influence of nicotinic agonists (e.g., nicotine patch) (Acheson et al. 2006; McKee et al. 2008), partial agonists (e.g., varenicline [Chantix^®^]) (Steensland et al. 2007), and antagonists (mecamylamine) (Blomqvist et al. 2002; Chi and de Wit 2003) on alcohol effects and alcohol consumption. These research findings suggest that all three classes of agents may show promise. There is considerable interest in the potential use of varenicline, a partial agonist at the α_4_β_2_ nicotinic receptor and a full agonist at the α_7_ receptor that is approved by the FDA for smoking cessation treatment. Although this medication has been found to be relatively well tolerated for smoking cessation, recent FDA alerts indicate that it may produce increased suicidal ideation, suicidal behavior, and drowsiness. The potential use of this agent for the treatment of alcohol use stems from evidence suggesting that varenicline reduces alcohol drinking in rodent models (Steensland et al. 2007). Clinical trials and human laboratory studies of this agent for alcohol drinking are underway, but results are not yet available.

### CRF Antagonists

Corticotrophin-releasing factor (CRF) is a neuropeptide that has been shown to play an integral role in mediating stress responses. The link between stress and alcohol consumption has been widely studied. Stress increases alcohol use and is a significant trigger in relapse to drinking (see review by Sinha 2007). Preclinical studies suggest that CRF may be involved in alcohol self-administration during withdrawal (Valdez et al. 2002) and that CRF antagonists block alcohol withdrawal–induced anxiety (Baldwin et al. 1991). Moreover, levels of CRF1 receptors in dependent animals are increased following alcohol withdrawal, and CRF1 antagonists have been shown to reduce alcohol self-administration (for a review, see Heilig and Koob 2007). All the above evidence suggests that the CRF system may be implicated in stress-induced relapse to alcohol drinking and that CRF antagonists may be effective in treating alcohol use. However, few agents that target this system are available for human use, and the utility of existing antagonists has been questioned by recent evidence suggesting that they are not effective in the treatment of depression (Binneman et al. 2008).

### Neurokinin 1 Antagonists

Substance P, a neurotransmitter from the tachykinin family of neuropeptide receptors that is released in response to stress, preferentially binds to the neurokinin 1 (NK1) receptors. Because alcohol consumption has been shown to reduce stress, recent studies have focused on examining the relationship between alcohol use and the substance P system. Researchers found reduced levels of substance P in rats that preferentially consume alcohol (Slawecki et al. 2001). Mice that lack the NK1 receptor have been found to consume lower quantities of alcohol compared with control animals, and in a human laboratory model, an antagonist of neurokinin 1, LY686017, was found to reduce spontaneous as well as stress-and alcohol-cue–induced craving for alcohol in alcohol-dependent individuals (George et al. 2008). Clinical trials of agents that target this system are not yet available.

## Summary

Alcohol has a complex neuropharmacology and can affect many different brain neurotransmitter systems. Several pharmacological agents that interact with specific neurotransmitter systems affected by alcohol already have shown efficacy in the treatment of alcohol dependence and many exciting investigational agents are on the horizon. The development of these agents has been based on translational approaches ranging from the use of molecular techniques to understand alcohol neurobiology and identify candidate molecules, to the use of numerous animal models of alcohol-related behaviors to test the use and mechanisms of action underlying these agents, and finally the use of human clinical trials and laboratory paradigms to evaluate the efficacy of these agents. The figure provides an overview of the medications discussed in this review and their proposed sites of action on the neural pathways that have been shown to mediate alcohol reinforcement.

Understanding the neuroscience underlying alcohol reinforcement has served as the foundation for the development of these medications. Future research needs to focus on validating the effects of existing agents and examining genetic and patient-specific predictors of treatment response. Developing a better understanding of the pharmacogenetics of treatment response could lead to appropriate treatment matching and efficient utilization of existing treatment resources and medications. Future studies also need to extend these findings to subpopulations of alcoholics, such as those with comorbid psychiatric conditions. Although preliminary evidence seems to indicate beneficial effects of specific medications for such subgroups, this evidence should be replicated and further understood. Finally, because only modest efficacy has been observed with most existing agents and genetic studies clearly indicate that alcoholism has multiple genetic profiles, future clinical trials should consider adaptive designs in which nonresponders to one pharmacotherapy are either switched to or augmented with an alternative pharmacotherapy that targets a different neurotransmitter system.

## Figures and Tables

**Figure:**
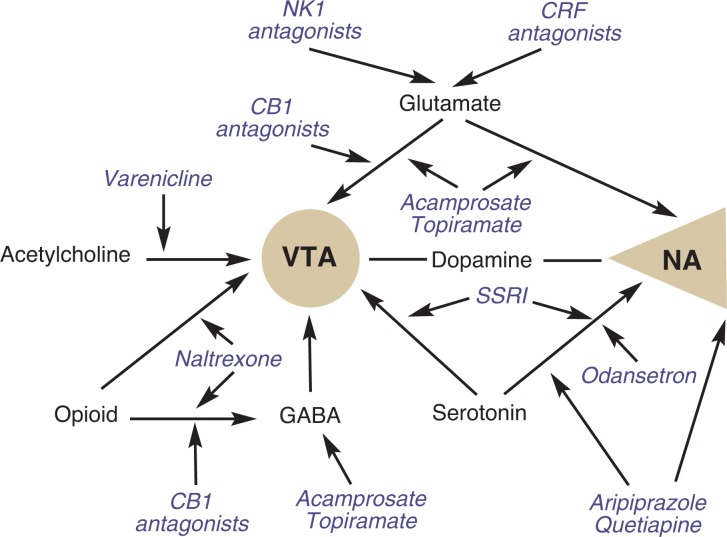
Medications and their proposed sites of action on the neural pathways that have been shown to mediate alcohol reinforcement. CB1 = cannabi-noid-1, CRF = corticotrophin-releasing factor, GABA = γ-aminobutyric acid, NA = nucleus accumbens, NK1 = neurokinin 1, VTA = ventral tegmental area.

## Footnotes

1 Definitions of this and other technical terms can be found in the Glossary, pp. 345–347.

## References

[b1-arh-31-4-400] Acheson A, Mahler SV, Chi H, de Wit H (2006). Differential effects of nicotine on alcohol consumption in men and women. Psychopharmacology.

[b2-arh-31-4-400] Addolorato G, Caputo F, Capristo E (2002). Baclofen efficacy in reducing alcohol craving and intake: A preliminary double-blind randomized controlled study. Alcohol and Alcoholism.

[b3-arh-31-4-400] Addolorato G, Leggio L, Ferrulli A (2007). Effectiveness and safety of baclofen for maintenance of alcohol abstinence in alcohol-dependent patients with liver cirrhosis: Randomised, double-blind controlled study. Lancet.

[b4-arh-31-4-400] Anton RF, Kranzler H, Breder C (2008b). A randomized, multicenter, double-blind, placebo-controlled study of the efficacy and safety of aripiprazole for the treatment of alcohol dependence. Journal of Clinical Psychopharmacology.

[b5-arh-31-4-400] Anton RF, O’Malley SS, Ciraulo DA, COMBINE Study Research Group (2006). Combined pharmacotherapies and behavioral interventions for alcohol dependence: The COMBINE study: A randomized controlled trial. JAMA Journal of the American Medical Association.

[b6-arh-31-4-400] Anton RF, Oroszi G, O’Malley S (2008a). An evaluation of muopioid receptor (OPRM1) as a predictor of naltrexone response in the treatment of alcohol dependence: Results from the Combined Pharmacotherapies and Behavioral Interventions for Alcohol Dependence (COMBINE) study. Archives of General Psychiatry.

[b7-arh-31-4-400] Baldwin HA, Rassnick S, Rivier J (1991). CRF antagonist reverses the “anxiogenic” response to ethanol withdrawal in the rat. Psychopharmacology.

[b8-arh-31-4-400] Barnes NM, Sharp T (1999). A review of central 5-HT receptors and their function. Neuropharmacology.

[b9-arh-31-4-400] Binneman B, Feltner D, Kolluri S (2008). A 6-week randomized, placebo-controlled trial of CP-316,311 (a selective CRH1 antagonist) in the treatment of major depression. American Journal of Psychiatry.

[b10-arh-31-4-400] Blomqvist O, Hernandez-Avila CA, Van Kirk J (2002). Mecamylamine modifies the pharmacokinetics and reinforcing effects of alcohol. Alcoholism: Clinical and Experimental Research.

[b11-arh-31-4-400] Bouza C, Angeles M, Muñoz A, Amate JM (2004). Efficacy and safety of naltrexone and acamprosate in the treatment of alcohol dependence: A systematic review. Addiction.

[b12-arh-31-4-400] Carroll KM, Nich C, Ball SA (1998). Treatment of cocaine and alcohol dependence with psychotherapy and disulfiram. Addiction.

[b13-arh-31-4-400] Chi H, de Wit H (2003). Mecamylamine attenuates the subjective stimulant-like effects of alcohol in social drinkers. Alcoholism: Clinical and Experimental Research.

[b14-arh-31-4-400] Colombo G, Addolorato G, Agabio R (2004). Role of GABA(B) receptor in alcohol dependence: Reducing effect of baclofen on alcohol intake and alcohol motivational properties in rats and amelioration of alcohol withdrawal syndrome and alcohol craving in human alcoholics. Neurotoxicity Research.

[b15-arh-31-4-400] Colombo G, Serra S, Brunetti G (2002). Stimulation of voluntary ethanol intake by cannabinoid receptor agonists in ethanol-preferring sP rats. Psychopharmacology.

[b16-arh-31-4-400] Cornelius JR, Salloum IM, Ehler JG (1997). Fluoxetine in depressed alcoholics: A double-blind, placebo-controlled trial. Archives of General Psychiatry.

[b17-arh-31-4-400] Czachowski CL, Legg BH, Samson HH (2001). Effects of acamprosate on ethanol-seeking and self-administration in the rat. Alcoholism: Clinical and Experimental Research.

[b18-arh-31-4-400] Dahchour A, De Witte P, Bolo N (1998). Central effects of acamprosate: Part 1. Acamprosate blocks the glutamate increase in the nucleus accumbens microdialysate in ethanol withdrawn rats. Psychiatry Research.

[b19-arh-31-4-400] De Witte P, Littleton J, Parot P, Koob G (2005). Neuroprotective and abstinence-promoting effects of acamprosate: Elucidating the mechanism of action. CNS Drugs.

[b20-arh-31-4-400] Dyr W, Ligieza J, Kostowski W (2008). The effect of cannabinoid CB(1) receptor antagonist rimonabant (SR-141716) on ethanol drinking in high-preferring rats. Alcohol.

[b21-arh-31-4-400] Egli M (2005). Can experimental paradigms and animal models be used to discover clinically effective medications for alcoholism?. Addiction Biology.

[b22-arh-31-4-400] Farook JM, Morrell DJ, Lewis B (2007). Topiramate (Topamax) reduces conditioned abstinence behaviours and handling-induced convulsions (HIC) after chronic administration of alcohol in Swiss-Webster mice. Alcohol and Alcoholism.

[b23-arh-31-4-400] Flannery BA, Garbutt JC, Cody MW (2004). Baclofen for alcohol dependence: A preliminary open-label study. Alcoholism: Clinical and Experimental Research.

[b24-arh-31-4-400] Froehlich J, O’Malley SS, Hyytiä P (2003). Preclinical and clinical studies on naltrexone: What have they taught each other?. Alcoholism: Clinical and Experimental Research.

[b25-arh-31-4-400] Fuller RK, Branchey L, Brightwell DR (1986). Disulfiram treatment of alcoholism: A Veterans Administration cooperative study. JAMA: Journal of the American Medical Association.

[b26-arh-31-4-400] Garbutt JC, Kranzler HR, O’Malley SS, Vivitrex Study Group (2005). Efficacy and tolerability of long-acting injectable naltrexone for alcohol dependence: A randomized controlled trial. JAMA: Journal of the American Medical Association.

[b27-arh-31-4-400] Gass JT, Olive MF (2008). Glutamatergic substrates of drug addiction and alcoholism. Biochemical Pharmacology.

[b28-arh-31-4-400] Gelernter J, Gueorguieva R, Kranzler HR, VA Cooperative Study #425 Study Group (2007). Opioid receptor gene (OPRM1, OPRK1, and OPRD1) variants and response to naltrexone treatment for alcohol dependence: Results from the VA Cooperative Study. Alcoholism: Clinical and Experimental Research.

[b29-arh-31-4-400] George DT, Gilman J, Hersh J (2008). Neurokinin 1 receptor antagonism as a possible therapy for alcoholism. Science.

[b30-arh-31-4-400] Goldstein M, Nakajima K (1967). The effect of disulfiram on catecholamine levels in the brain. Journal of Pharmacology and Experimental Therapeutics.

[b31-arh-31-4-400] Goldstein M, Anagnoste B, Lauber E, Mckeregham MR (1964). Inhibition of dopamine-beta-hydroxylase by disulfiram. Life Sciences.

[b32-arh-31-4-400] Gorelick DA, Paredes A (1992). Effect of fluoxetine on alcohol consumption in male alcoholics. Alcoholism: Clinical and Experimental Research.

[b33-arh-31-4-400] Gueorguieva R, Wu R, Pittman B (2007). New insights into the efficacy of naltrexone based on trajectory-based reanalyses of two negative clinical trials. Biological Psychiatry.

[b34-arh-31-4-400] Gulley JM, McNamara C, Barbera TJ (1995). Selective serotonin reuptake inhibitors: Effects of chronic treatment on ethanol-reinforced behavior in mice. Alcohol.

[b35-arh-31-4-400] Harris BR, Prendergast MA, Gibson DA (2002). Acamprosate inhibits the binding and neurotoxic effects of trans-ACPD, suggesting a novel site of action at metabotropic glutamate receptors. Alcoholism: Clinical and Experimental Research.

[b36-arh-31-4-400] Heilig M, Koob GF (2007). A key role for corticotropin-releasing factor in alcohol dependence. Trends in Neurosciences.

[b37-arh-31-4-400] Ingman K, Kupila J, Hyytiä P, Korpi ER (2006). Effects of aripiprazole on alcohol intake in an animal model of high-alcohol drinking. Alcohol and Alcoholism.

[b38-arh-31-4-400] Johnson BA, Campling GM, Griffiths P, Cowen PJ (1993). Attenuation of some alcohol-induced mood changes and the desire to drink by 5-HT3 receptor blockade: A preliminary study in healthy male volunteers. Psychopharmacology.

[b39-arh-31-4-400] Johnson BA, Roache JD, Javors MA (2000). Ondansetron for reduction of drinking among biologically predisposed alcoholic patients: A randomized controlled trial. JAMA: Journal of the American Medical Association.

[b40-arh-31-4-400] Johnson BA, Rosenthal N, Capece JA, Topiramate for Alcoholism Advisory Board, Topiramate for Alcoholism Study Group (2008). Improvement of physical health and quality of life of alcohol-dependent individuals with topiramate treatment: US multisite randomized controlled trial. Archives of Internal Medicine.

[b41-arh-31-4-400] Kampman KM, Pettinati HM, Lynch KG (2007). A double-blind, placebo-controlled pilot trial of quetiapine for the treatment of Type A and Type B alcoholism. Journal of Clinical Psychopharmacology.

[b42-arh-31-4-400] Kranzler HR, Gage A (2008). Acamprosate efficacy in alcohol-dependent patients: Summary of results from three pivotal trials. American Journal on Addictions.

[b43-arh-31-4-400] Kranzler HR, Burleson JA, Korner P (1995). Placebo-controlled trial of fluoxetine as an adjunct to relapse prevention in alcoholics. American Journal of Psychiatry.

[b44-arh-31-4-400] Kranzler HR, Burleson JA, Brown J, Babor TF (1996). Fluoxetine treatment seems to reduce the beneficial effects of cognitive-behavioral therapy in type B alcoholics. Alcoholism: Clinical and Experimental Research.

[b45-arh-31-4-400] Kranzler HR, Pierucci-Lagha A, Feinn R, Hernandez-Avila C (2003). Effects of ondansetron in early-versus late-onset alcoholics: A prospective, open-label study. Alcoholism: Clinical and Experimental Research.

[b46-arh-31-4-400] Kranzler HR, Mueller T, Cornelius J (2006). Sertraline treatment of co-occurring alcohol dependence and major depression. Journal of Clinical Psychopharmacology.

[b47-arh-31-4-400] Kranzler HR, Covault J, Pierucci-Lagha A (2008). Effects of aripiprazole on subjective and physiological responses to alcohol. Alcoholism: Clinical and Experimental Research.

[b48-arh-31-4-400] Krishnan-Sarin S, Krystal JH, Shi J (2007). Family history of alcoholism influences naltrexone-induced reduction in alcohol drinking. Biological Psychiatry.

[b49-arh-31-4-400] Krupitsky EM, Rudenko AA, Burakov AM (2007). Antiglutamatergic strategies for ethanol detoxification: Comparison with placebo and diazepam. Alcoholism: Clinical and Experimental Research.

[b50-arh-31-4-400] Krystal JH, Cramer JA, Krol WF, Veterans Affairs Naltrexone Cooperative Study 425 Group (2001). Naltrexone in the treatment of alcohol dependence. New England Journal of Medicine.

[b51-arh-31-4-400] Lê AD, Wang A, Harding S (2003). Nicotine increases alcohol self-administration and reinstates alcohol seeking in rats. Psychopharmacology.

[b52-arh-31-4-400] Littleton J, Zieglgänsberger W (2003). Pharmacological mechanisms of naltrexone and acamprosate in the prevention of relapse in alcohol dependence. American Journal on Addictions.

[b53-arh-31-4-400] Löf E, Olausson P, deBejczy A (2007). Nicotinic acetylcholine receptors in the ventral tegmental area mediate the dopamine activating and reinforcing properties of ethanol cues. Psychopharmacology.

[b54-arh-31-4-400] Lovinger DM, Crabbe JC (2005). Laboratory models of alcoholism: Treatment target identification and insight into mechanisms. Nature Neuroscience.

[b55-arh-31-4-400] Marinelli PW, Quirion R, Gianoulakis C (2003). A microdialysis profile of beta-endorphin and catecholamines in the rat nucleus accumbens following alcohol administration. Psychopharmacology.

[b56-arh-31-4-400] Mason BJ, Goodman AM, Chabac S, Lehert P (2006). Effect of oral acamprosate on abstinence in patients with alcohol dependence in a double-blind, placebo-controlled trial: The role of patient motivation. Journal of Psychiatric Research.

[b57-arh-31-4-400] Mann K, Lehert P, Morgan MY (2004). The efficacy of acamprosate in the maintenance of abstinence in alcohol-dependent individuals: Results of a meta-analysis. Alcoholism: Clinical and Experimental Research.

[b58-arh-31-4-400] McKee SA, O’Malley SS, Shi J (2008). Effect of transdermal nicotine replacement on alcohol responses and alcohol self-administration. Psychopharmacology.

[b59-arh-31-4-400] Monnelly EP, Ciraulo DA, Knapp C (2004). Quetiapine for treatment of alcohol dependence. Journal of Clinical Psychopharmacology.

[b60-arh-31-4-400] Myers RD, Veale WL (1968). Alcohol preference in the rat: Reduction following depletion of brain serotonin. Science.

[b61-arh-31-4-400] Naassila M, Pierrefiche O, Ledent C, Daoust M (2004). Decreased alcohol self-administration and increased alcohol sensitivity and withdrawal in CB1 receptor knockout mice. Neuropharmacology.

[b62-arh-31-4-400] Naranjo CA, Bremner KE, Lanctôt KL (1995). Effects of citalopram and a brief psycho-social intervention on alcohol intake, dependence and problems. Addiction.

[b63-arh-31-4-400] Nguyen SA, Malcolm R, Middaugh LD (2007). Topiramate reduces ethanol consumption by C57BL/6 mice. Synapse.

[b64-arh-31-4-400] O’Malley SS, Jaffe AJ, Chang G (1992). Naltrexone and coping skills therapy for alcohol dependence: A controlled study. Archives of General Psychiatry.

[b65-arh-31-4-400] O’Malley SS, Krishnan-Sarin S, Farren C (2002). Naltrexone decreases craving and alcohol self-administration in alcohol-dependent subjects and activates the hypothalamo-pituitary-adrenocortical axis. Psychopharmacology.

[b66-arh-31-4-400] Oslin DW, Berrettini W, Kranzler HR (2003). A functional polymorphism of the mu-opioid receptor gene is associated with naltrexone response in alcohol-dependent patients. Neuropsychopharmacology.

[b67-arh-31-4-400] Petrakis IL, Poling J, Levinson C, VA New England VISN I MIRECC Study Group (2005). Naltrexone and disulfiram in patients with alcohol dependence and comorbid psychiatric disorders. Biological Psychiatry.

[b68-arh-31-4-400] Pettinati HM, Volpicelli JR, Kranzler HR (2000). Sertraline treatment for alcohol dependence: Interactive effects of medication and alcoholic subtype. Alcoholism: Clinical and Experimental Research.

[b69-arh-31-4-400] Reilly MT, Lobo IA, McCracken LM (2008). Effects of acamprosate on neuronal receptors and ion channels expressed in Xenopus oocytes. Alcoholism: Clinical and Experimental Research.

[b70-arh-31-4-400] Shapiro DA, Renock S, Arrington E (2003). Aripiprazole, a novel atypical antipsychotic drug with a unique and robust pharmacology. Neuropsychopharmacology.

[b71-arh-31-4-400] Sinha R (2007). The role of stress in addiction relapse. Current Psychiatry Reports.

[b72-arh-31-4-400] Slawecki CJ, Jimenéz-Vasquez P, Mathé AA, Ehlers CL (2001). Substance P and neurokinin levels are decreased in the cortex and hypothalamus of alcohol-preferring (P) rats. Journal of Studies on Alcohol.

[b73-arh-31-4-400] Soyka M, Koller G, Schmidt P, ACTOL Study Investigators (2008). Cannabinoid receptor 1 blocker rimonabant (SR 141716) for treatment of alcohol dependence: Results from a placebo-controlled, double-blind trial. Journal of Clinical Psychopharmacology.

[b74-arh-31-4-400] Spanagel R, Putzke J, Stefferl A (1996). Acamprosate and alcohol: II. Effects on alcohol withdrawal in the rat. European Journal of Pharmacology.

[b75-arh-31-4-400] Srisurapanont M, Jarusuraisin N (2002). Opioid antagonists for alcohol dependence. Cochrane Database System Reviews.

[b76-arh-31-4-400] Steensland P, Simms JA, Holgate J (2007). Varenicline, an alpha4beta2 nicotinic acetylcholine receptor partial agonist, selectively decreases ethanol consumption and seeking. Proceedings of the National Academy of Sciences of the United States of America.

[b77-arh-31-4-400] Swift RM (2000). Opioid antagonists and alcoholism treatment. CNS Spectrums.

[b78-arh-31-4-400] Swift RM, Davidson D, Whelihan W, Kuznetsov O (1996). Ondansetron alters human alcohol intoxication. Biological Psychiatry.

[b79-arh-31-4-400] Valdez GR, Roberts AJ, Chan K (2002). Increased ethanol self-administration and anxiety-like behavior during acute ethanol withdrawal and protracted abstinence: Regulation by corticotropin-releasing factor. Alcoholism: Clinical and Experimental Research.

[b80-arh-31-4-400] Verheul R, Lehert P, Geerlings PJ (2005). Predictors of acamprosate efficacy: Results from a pooled analysis of seven European trials including 1485 alcohol-dependent patients. Psychopharmacology.

[b81-arh-31-4-400] Volpicelli JR, Alterman AI, Hayashida M, O’Brien CP (1992). Naltrexone in the treatment of alcohol dependence. Archives of General Psychiatry.

[b82-arh-31-4-400] Zalewska-Kaszubska J, Gorska D, Dyr W, Czarnecka E (2006). Effect of acute administration of ethanol on beta-endorphin plasma level in ethanol preferring and non-preferring rats chronically treated with naltrexone. Pharmacology, Biochemistry, and Behavior.

